# Tunable Photoluminescence Properties of Cotton Fiber With Gradually Changing Crystallinity

**DOI:** 10.3389/fchem.2022.805252

**Published:** 2022-06-28

**Authors:** Qing Zhou, Man Liu, Chuchu Li, Shijia Lu, Bin Lei, Jiantang Jiang, Ying Yin, Yuanchao Zhang, Yifeng Shen

**Affiliations:** ^1^ Engineering Research Center for Eco-Dyeing and Finishing of Textiles, Key Laboratory of Advanced Textile Materials and Manufacturing Technology, Ministry of Education, College of Textile Science and Engineering (International Institute of Silk), Zhejiang Sci-Tech University, Hangzhou, China; ^2^ Dali Silk (Zhejiang) Co., Ltd., Dali Science and Technology Park, Nanyan Provincial High-tech Development Zone, Shaoxing, China

**Keywords:** cotton fiber, crystallinity, nonconventional luminogens, persistent room temperature phosphorescence, clustering-triggered emission

## Abstract

The alkali mercerizing process of semicrystalline cotton fiber (CF) is widely used in the printing and dyeing industry. The crystallinity change in the mercerizing process has been studied and certain laws have been obtained, but there is still a certain distance between the theoretical research results and the practical applications. CF is almost composed of cellulose, combined with the photoluminescence (PL) phenomenon of cellulose; herein, the varying crystallinity is correlated with its PL behavior after being treated with different concentrations of NaOH. In line with the characteristics of nonconventional luminogens, CF enjoys excitation-dependent emission and persistent room temperature phosphorescence (p-RTP) behavior. The emission spectra of all samples under the same excitation wavelength indicate that the change of CF crystallinity has a significant impact on its fluorescence and p-RTP emission. As the concentration of NaOH increases, the varying trend of quantum efficiency (QY) is consistent with the changed crystallinity of CF. Interestingly, the lifetime of p-RTP is exactly the opposite of the crystallinity change law. Clustering-triggered emission (CTE), crystallization-Induced Phosphorescence (CIP) mechanism, and the swelling due to hydrated sodium ions can reasonably explain these interesting photophysical processes, which also can be supported by theoretical calculations. The above studies have basically clarified the inherent law between the crystalline change of CF and the PL emission behavior during the alkali treatment process, which can be used as a theoretical reference for real-time monitoring of CF crystallinity changes using the spectral method in the actual cotton mercerizing process.

## Introduction

Cotton fiber (CF) is one of the most common fibers in the textile printing and dyeing industry. In order to obtain excellent gloss and dyeing properties, CF will be alkali-treated through the alkali mercerizing process (Yazdanshenas and Shateri-Khalilabad, 2013; Ramamoorthy et al., 2015; Rajkumar et al., 2016). The cellulose content of CF is more than 90%, which is the source of natural cellulose with the highest purity (Abidi et al., 2014; Moon et al., 2011; Roche et al., 1978). Generally, after the CF is alkali mercerized, due to the fiber puffing, the light reflection behavior of CF is more regular, thus enhancing the luster (Akerholm et al., 2004). At the same time, the increase of the amorphous fixed area of the fiber increases the dye uptake rate during dyeing (Tsuboi, 1957; Okano and Sarko, 1985; Samsudin et al., 2020). In the current process, in addition to some research on the online control system of alkali concentration in the mercerizing process, there is almost no means to accurately monitor the crystallinity conversion of CF during the mercerizing process (Ujhelyiova et al., 2007). In case, sensitive monitoring methods such as light or electricity can be used to accurately control the entire mercerizing process to achieve fiber quality, which is very meaningful. The photoluminescence (PL) property of cellulose discovered in recent years has brought hope and feasibility to the abovementioned solutions (Gong et al., 2013; Du et al., 2019; Jiang et al., 2021; Lee et al., 2004). In 2013, Yuan et al. reported the luminescence behavior of natural polymers such as rice, starch, and cellulose, and found that they can emit bright light under ultraviolet (UV) light, and proposed a clustering-triggered emission mechanism (CTE), namely, the clustering of nonconventional chromophores with π and n electrons, and subsequent through space conjugation result in extended electron delocalization and conformation rigidification, to rationalize the emission, and to explain their intrinsic luminescence (Gong et al., 2013). In 2019, Du et al. (2019)found that microcrystalline cellulose (MCC) and its derivatives have room temperature phosphorescence (RTP) emission and used the CTE mechanism to explain such emission behavior. These substances also exhibit aggregation-induced emission (AIE) property (Dong et al., 2022).

Moreover, the luminescent compounds poly(amidoamine)s (PAMAM) (Lee et al., 2004; Lin et al., 2011; Lu et al., 2015), poly(amino ester)s (PAE) (Wu et al., 2005), poly(ether amide)s (PEA) (Lin et al., 2009), polyethylenimines (PEI) (Pastor-Pérez et al., 2007), and peptides, as (Guan et al., 2017) reported earlier, should be able to explain their luminescence behavior using the CTE mechanism. Subsequently, many researchers used the CTE mechanism to explain a series of newly discovered non-conjugated luminescence phenomena, such as Xylitol (Wang et al., 2018), non-conjugated amino acid (Chen et al., 2018), 1,1,2,2-tetraphenylethane (Zhang et al., 2017), and MDM2 proteins (Liu et al., 2020), this mechanism has been recognized by more and more researchers. Furthermore, persistent room temperature phosphorescence (p-RTP) phenomenon has been observed from many non-conjugated organic compounds, such as poly(acrylic acid) (PAA) (Zhou et al., 2020), polyacrylamide (PAM) (Wang et al., 2020), cyanoacetic acid (Fang et al., 2018), sodium polymethacrylate (PMANa) (Cai et al., 2019), and oxalic acid (Zheng et al., 2020). Moreover, non-conjugated luminescent compounds have a unique excitation wavelength dependence emission property, generally RTP phenomenon and its wide application prospects in multiple anti-counterfeiting and encryption fields have become research hotspots (Liao et al., 2021a; Xu et al., 2021a; Liao et al., 2021b; Xu et al., 2021b). However, at present, the phosphorescence ability of such compounds is weak at room temperature, and it can even be observed only in a vacuum for some compounds, for example, BSA (Wang et al., 2019). In order to enhance the p-RTP emission of such materials it may be possible to learn from the currently more recognized crystallization-induced phosphorescence (CIP) mechanism (Yuan et al., 2010; Shen et al., 2017; Chen et al., 2019; Nitti et al., 2020; Xing et al., 2020), that is, to build a rigid environment by increasing crystallization to inhibit non-radiative transitions (Sudhakar and Radhakrishnan, 2019; Yang et al., 2020). Therefore, the change in crystallinity has a significant impact on its luminescence behavior.

In order to explore the changes in the crystalline structure of CFs during alkali treatment (Supplementary Figure S1), the PL detection method is combined with crystallinity changes, so that it can macroscopically characterize the changes in the mercerization process of CFs through luminescence. In order to obtain a variety of CFs with adjustable crystallinity, we use traditional NaOH treatment solutions that cannot be concentrated. A series of CF samples with different crystallinity were prepared *via* different concentrations of NaOH aqueous solution, and the changes in crystallinity with luminescence behavior were carried out. The results showed that with the increase of alkali treatment concentration, the crystallinity of CF showed a trend of first decreasing and then increasing, and the QY change trend was the same as mentioned above. It is very interesting that in this process the p-RTP lifetime of CFs changes in the opposite direction to their crystallinity, and the most probable reason has been proven that the CF gradually swells due to hydrated sodium ions, resulting in the formation of more and stronger triplet emission centers.

## Results and Discussion

**FIGURE 1:**
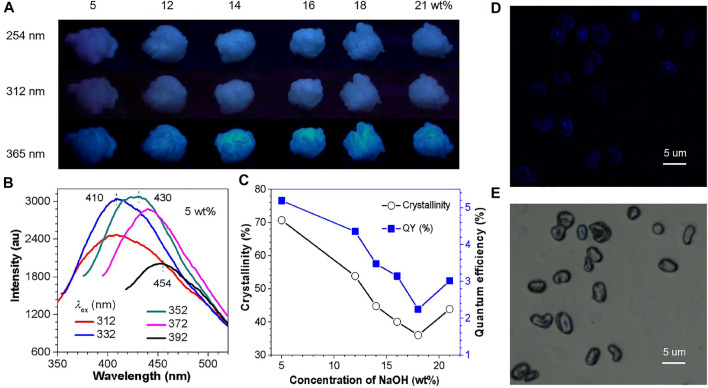
**(A)** Photographs of CF samples taken under varying UV lights (254, 312, 365 nm) at ambient conditions. **(B)** PL spectra of CF-5 with different *λ*_ex_ values. **(C)** Trend graph of crystallinity and QY (%) of samples. **(D)** Confocal image recorded under excitation at 405 nm of the CF cross-section and **(E)** bright field image.

As shown in Figure 1, the crystallinity of CF calculated from XRD firstly drops and then rises with the increased NaOH concentration (Supplementary Figure S2), and then reaches the lowest value at 18 wt% (36.01%) (Figure 1C, Supplementary Table S1). In the above process, the violently evolved crystallinity is very likely to change the structure of the cluster and the degree of conformation rigidification. Thus, according to the CTE and CIP theory, these changes will have a huge impact on the FL and p-RTP emission of CFs. In order to prove our guess, these CFs were investigated under different UV lamps (254, 312, and 365 nm). It can be seen that all the samples emit blue light under different UV lamps (Figure 1A), and the excitation spectra of the emission peak at 410 nm shows that the best excitation of these samples is around 352 nm (Supplementary Figure S3). Furthermore, these samples have excitation-dependent emission under different excitation (Figure 1B, Supplementary Figure S4), which implied the existence of multiple launch centers.

The QY data also revealed a higher consistency of crystallinity, indicating that crystallization can contribute to improve QY (Figure 1C). Specifically, the CF-5 enjoys the highest crystallinity and QY (70.70%, 5.19%), and the crystallinity and QY of CF-18 reached the lowest value (36.01%, 2.24%), while the crystallinity and QY of CF-21 increased simultaneously (43.86%, 3.01%) (Table S1). The above observations are macroscopic, and the nature of the macro is always determined by the micro structure. Thus, do the internal cross-sections of CF have similar phenomena? The image of the radial cross-section of the CF taken by the laser confocal microscope shows that the inside also has a relatively strong PL behavior (Figures 1D,E). Moreover, the PL emission phenomenon of CFs observed in a microscopic scale has hardly been reported.

**FIGURE 2:**
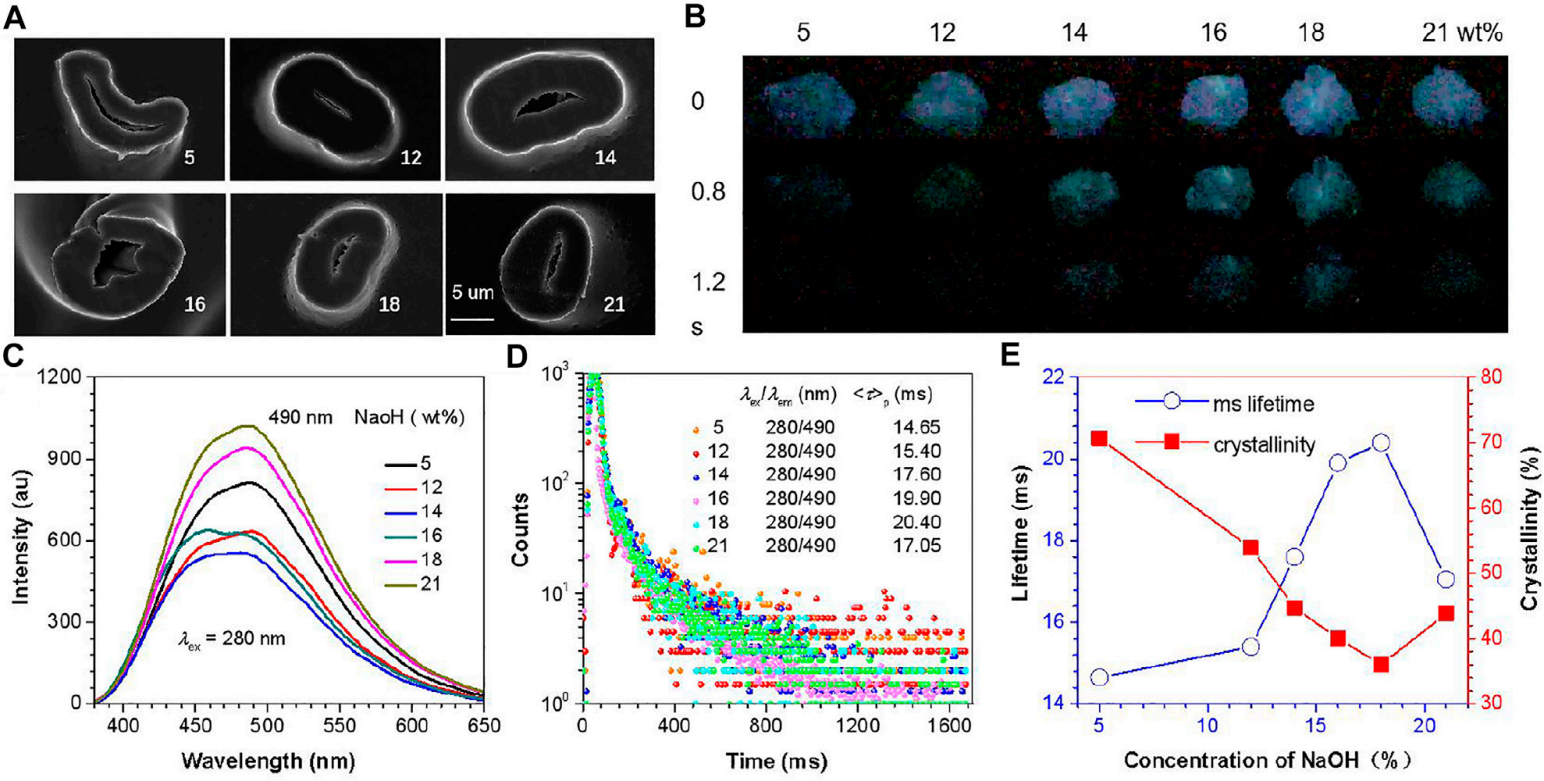
**(A)** SEM images of CF cross-sections with different crystallinity (scale bar = 5 um). **(B)** Photographs of samples after ceasing the 254 nm irradiation at ambient conditions. **(C)** Phosphorescence of CF sample at excitation wavelength of 280 nm (*t*_d_ = 0.1 ms). **(D)** Phosphorescence ms lifetimes of different fiber samples (*λ*_ex_ = 280 nm). **(E)** Change trend of ms lifetime and crystallinity of samples.

In the process of alkali treatment, the crystallinity also changes the morphology of the fiber. This can be seen from the SEM images of the cross-sections of these samples (Figure 2A). As the alkali treatment concentration increases, the CF gradually swells. The type changes to an elliptical cross-section, which is caused by the swelling of the amorphous region and the transformation of the crystalline region into the amorphous region and the realignment of the molecules. In the above morphological changes, the more important thing is that the changes in molecular arrangement will lead to change in intermolecular interactions, which will affect various physical parameters of CFs, especially the changes in the structure of the cluster emission center, thereby changing its PL behavior. Combining CIP theory and CTE mechanism, changes in crystallinity are likely to affect RTP emission property. Unsurprisingly, after turning off the UV lamp of the same wavelength, the p-RTP emission of samples with different crystallinity have a certain difference in emission peaks and lifetime (Figures 2B,C, Supplementary Figure S5 and Supplementary Video S1), which indicates that the emission ability of the emission center is different. The ms lifetimes are greatly increased as the crystallinity decreases until CF-18 (Figures 2D,E, Supplementary Table S2), and then it increased, basically contrary to the changing trend of crystallinity.

According to the mechanism of CTE and CIP, the increase of crystallinity should increase the emission ability of fluorescence and p-RTP. In general, the arrangement of cellulose molecules in the crystalline region must be denser than that in the amorphous region, resulting in a closer average distance between oxygen atoms in the crystalline region. It is easier for electrons to be delocalized and conjugated to form cluster emission centers with strong emission ability. With the increase of crystallinity, the number and emission ability of the cluster also increase. The above finding can be reasonably understood by the CTE mechanism. The increase of crystallinity makes the distance between some other atoms closer, which leads to the increase of interaction and the rigidity of the conformation of the cluster, and improves the radiation transition probability, which can be reasonably explained by the CIP mechanism.

**FIGURE 3:**
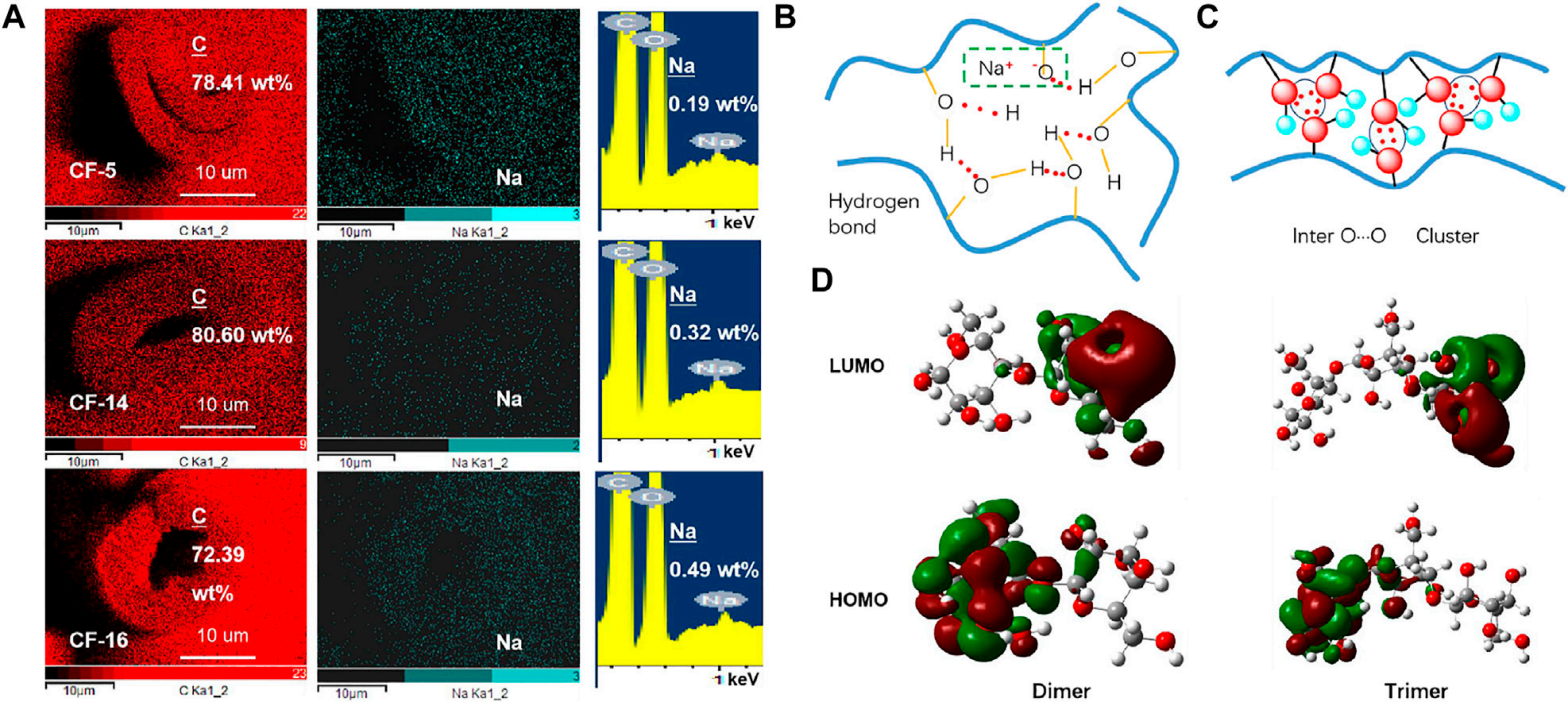
**(A)** SEM mapping images of CF cross-sections with different crystallinity (scale bar = 10 um), area scans of C and Na elemental distribution. **(B)** Partial hydrogen bond interactions in CFs. **(C)** The oxygen clusters in CFs are mainly formed by the interaction of oxygen atoms. **(D)** LUMO and HOMO electron densities of the dimer and trimer.

The change of QY emission ability and the change of crystallinity can support the above conclusion, but p-RTP has the opposite conclusion which is probably because when the crystallinity increases, the per unit volume of CF also increases, and the volume of air decreases, which is defined as the swelling effect (Figure 3A and Supplementary Figure S6). This effect has been recognized as a phenomenon, and its mechanism is generally believed to be derived from the destruction of the lattice and the reconstruction of bonds by hydrated sodium ions (Gert et al., 2000). Because of the presence of Na^+^ ions, which apparently bond with the cellulose hydroxyl groups, almost all of the interchain hydrogen bonds that ordinarily stabilize the cellulose structure have been broken. New types of interchain bonds were formed by the help of Na^+^ ions and water molecules present in the system (Klemm et al., 1998). Sodium ions partially remain in the amorphous region due to ionic interactions with oxygen anions, and its content increase with the increase of the amorphous region which can be supported from their mapping elements (carbon, oxygen, and sodium) results calculated by deducting the background and integral area (Figure 3A, Supplementary S7–S9, Supplementary Table S3). The interaction of Na^+^ and O^−^ ions (removal of H from hydroxyl groups on cellulose) to form part of ionic bond greatly increases the molecular conformation rigidity. It can form an emission center with stronger triplet emission capability, so that the CF with low crystallinity has a longer afterglow instead.

The PL phenomenon of CF can be explained by the CTE mechanism, the clustering of oxygen atoms created enriched energy levels and narrowed the energy gaps, thus promoting SOC and allowing the consequent ISC transitions. It can be seen from the above analysis that the influence of crystallinity conversion process on the PL performance of CF has been partially obtained. How do the molecules in the CF affect its emission? In-depth analysis, it can be found that strong hydrogen bond interactions are formed between the hydroxyl groups on the cellulose molecular structure, especially in the crystalline region (Figure 3B). The remaining Na ions in the intermolecular and O atoms form partial ionic bonds, resulting in a more rigid cluster emission center. In addition, the interaction between the lone pair of oxygen atoms forms an oxygen cluster structure, which increases the rigidity of the cluster’s conformation under the action of hydrogen bonds, and makes it have PL ability. In order to further understand why the cellulose molecule has PL and p-RTP emission capability, the preliminary theoretical calculations were conducted. LUMO and HOMO electron densities of the dimer (the smallest unit of cellulose) and trimer of the cellulose unit were calculated, and their molecular conformations are optimized. Although the results remain preliminary, the electron density distribution clearly indicates the intramolecular O···O electron delocalization for the leftmost single molecule in the dimer and trimer (Figure 3D). In addition, the HOMO and LUMO levels of aggregates clearly illustrated the extended delocalization among the neighboring molecules in excited states, which agreed well with the postulated hypothesis.

## Conclusion

In conclusion, CF enjoys excitation-dependent emission and p-RTP behavior. Moreover, the emission spectra of all samples under the same excitation wavelength indicate that the change of CF crystallinity has a significant impact on its fluorescence and p-RTP emission. The increased QY is attributed to the increase in crystallinity, whereas the emission ability of p-RTP exhibits a negative correlation. These interesting phenomena can be reasonably explained by CTE and the swelling due to hydrated sodium ions. Furthermore, these findings, in turn, offer more fundamental implications to the underlying mechanism of nonconventional chromophores. The exploration of the CIP and CTE laws of CF is likely to fill the gap in the study of photoluminescence behavior in the process of crystallization conversion of the natural polymer (fiber). More meaningful is that these results can be used as a theoretical reference for real-time monitoring of CF or other natural fiber in the actual mercerizing process.

## Data Availability

The original contributions presented in the study are included in the article/Supplementary Material, further inquiries can be directed to the corresponding authors.
