# Supplementary material for: Tunable Photoluminescence Properties of Cotton Fiber With Gradually Changing Crystallinity
Source: Front Chem. 2022 Jun 28;10:805252. doi: 10.3389/fchem.2022.805252 (PMC9274137; doi:10.3389/fchem.2022.805252)
Supplement: Supplementary file 1 [file DataSheet1.pdf]

Supporting Information

Tunable photoluminescence properties of cotton fiber with gradually changing crystallinity and crystal form

Qing Zhou[1], Man Liu[1], Chuchu Li[1], Shijia Lu[1], Bin Lei[2], Jiantang Jiang[1], Ying Yin[1], Yuanchao Zhang[1], Yifeng Shen[1]**

[1]Engineering Research Center for Eco-Dyeing and Finishing of Textiles, Key Laboratory of Advanced Textile Materials and Manufacturing Technology, Ministry of Education, College of Textile Science and Engineering (International Institute of Silk), Zhejiang Sci-Tech University, 928 Second Avenue, Hangzhou, Zhejiang, 310018 China
[2] Dali Silk (Zhejiang) Co., Ltd., Dali Science and Technology Park, Nanyan Provincial High-tech Development Zone, Xinchang County, Shaoxing City, Shaoxing, Zhejiang, 310018 China
*** Correspondence:**
Qing Zhou, Yifeng Shen
E-mail: qingzhou1@zstu.edu.cn; syf@zstu.edu.cn

Methods

Materials. Sodium hydroxide (NaOH, 99%), which were purchased from Comeo Chemical Reagent Co., Ltd. De-seed virgin cotton fiber (produced in Aksu, Xinjiang), purchased from Shanghai Yuanjiang Chemical Co., Ltd., was further purified with 5 wt% NaOH solution for 24 h to remove impurity, and then washed with deionized water until PH reached 7.0. Purified water was purchased from Hangzhou Wahaha Group Co., Ltd and used as received.

Preparation of cotton fiber with different crystallinity Different mass fractions of NaOH solutions (12, 14, 16, 18 and 21 wt%) were used to treat cotton fiber in a water bath (25°C, 8

h). Rinse with deionized water until the pH value of the cotton fiber surface is 7.0, and dry the obtained cotton fiber in a vacuum oven at 50 °C.

Instrumentation. The solid state ^{13}C NMR spectra are acquired from a Bruker AVANCE III HD 600 MHz. XRD measurements were conducted on powders and films with a D8 Advance diffractometer (Bruker, Germany) and a Cu Kα radiation source (λ = 1.5418 Å), at a scanning rate of 6° min^{-1}. The photoluminescence (PL) spectroscopy of samples were obtained with the fluorescence spectrophotometer (F-46001, Japan). Delayed PL spectra of solids at room temperature and phosphorescence lifetimes were measured on an Edinburgh FLS980 fluorescence spectrometer. Photoluminescence quantum yields (PLQY) of solids were measured on Edinburgh FLS980 equipped with integrating sphere. The SEM mapping scanning elemental analysis was performed by X-MaxN instrument (Oxford). Luminescent photographs were taken with a SLR camera (Canon EOS 70D, Japan), and the videos were recorded using acamera (Sony A7S2, Japan) and the afterglow images were captured from the videos. The photoluminescence photos under the microscopic view are taken through a confocal laser scanning microscope (Nikon $C2^+$ System; Nikon Instruments Inc, Melville, NY).

Calculation the crystallinity of cotton fiber. The XRD curve of the CF is fitted by the Lorentzian function to obtain the crystalline diffraction peaks and amorphous peaks corresponding to characteristic peaks (2θ=12.1°, 14.8°, 16.6°, 20.1°, 22°, 22.6°) via MDI Jade 6.0 software, respectively. The crystallinity of the cellulose can be obtained by calculating the peak area by formula (1).[1,2]

$$\mathrm{CrI\%} = \frac{\mathrm{Sp}}{\mathrm{IA} + \mathrm{Sp}} \times 100\% \tag{1}$$

Here, IA is the integrated area of the amorphous region, and Sp is the total integrated area of the crystalline region.

Calculation of elemental content from EDX mapping data. OriginPro 2016 software is used to deduct the background of the original data and the curve after subtracting the background was obtained. The content of C\O\Na elements was obtained by the method of segmental integration of the peak range of elements.

Calculation of HOMO and LUMO Electron Densities. The further geometry optimization of coupling dimers and trimers structure were conducted in order to maintain the specific molecular configuration and the conformational search was not performed before performing a geometry optimization by Density Functional Theory (DFT). The Gaussian 09 program was utilized to perform the TD-DFT calculations of the dimer and trimer of cellulose with the opt b3lyp/6-31g(d) method. The HOMO and LUMO energy densities were obtained via GaussView 5.0.8 (isovalue-0.02, cube grid-coarse).

NMR Characterization

(CF-5) ^{13}C NMR (151 MHz) δ 101.80, 85.54, 80.53, 71.77, 68.86, 62.06, 58.61.

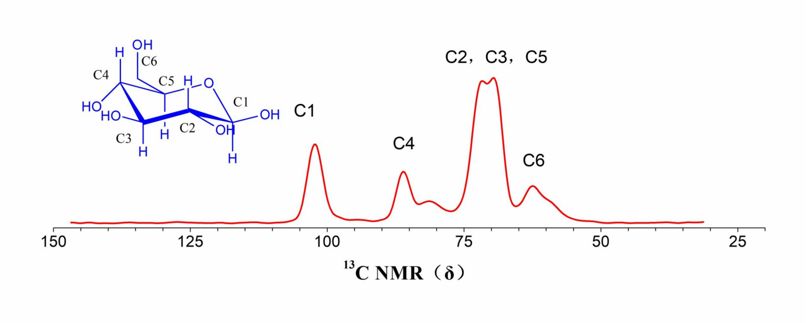

Figure S1. ^{13}C NMR spectra of 5 wt%.

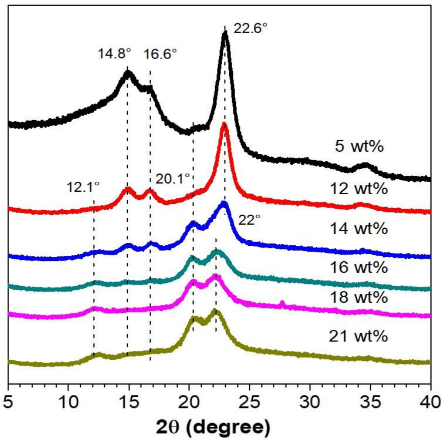

Figure S2. X-ray powder diffraction pattern of cotton fiber treated with different concentrations of NaOH (5, 12, 14, 16, 18, 21 wt%) solution.

Table S1. Changes of crystallinity and QY of samples after alkali treatment with different NaOH concentration.

Samples	NaOH (wt%)	Crystallinity (%)	QY (%)
CF-5	5	70.70	5.19
CF-12	12	53.85	4.36
CF-14	14	44.76	3.48
CF-16	16	40.05	3.15
CF-18	18	36.01	2.24
CF-21	21	43.86	3.01

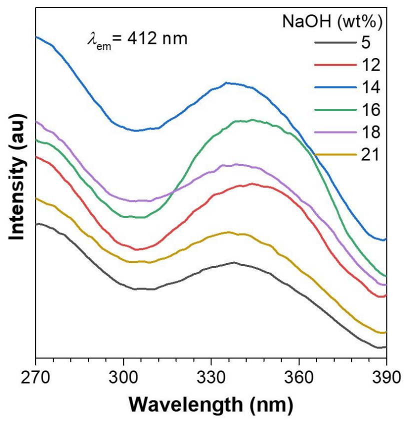

Figure S3. The excitation spectra of samples at different emission.

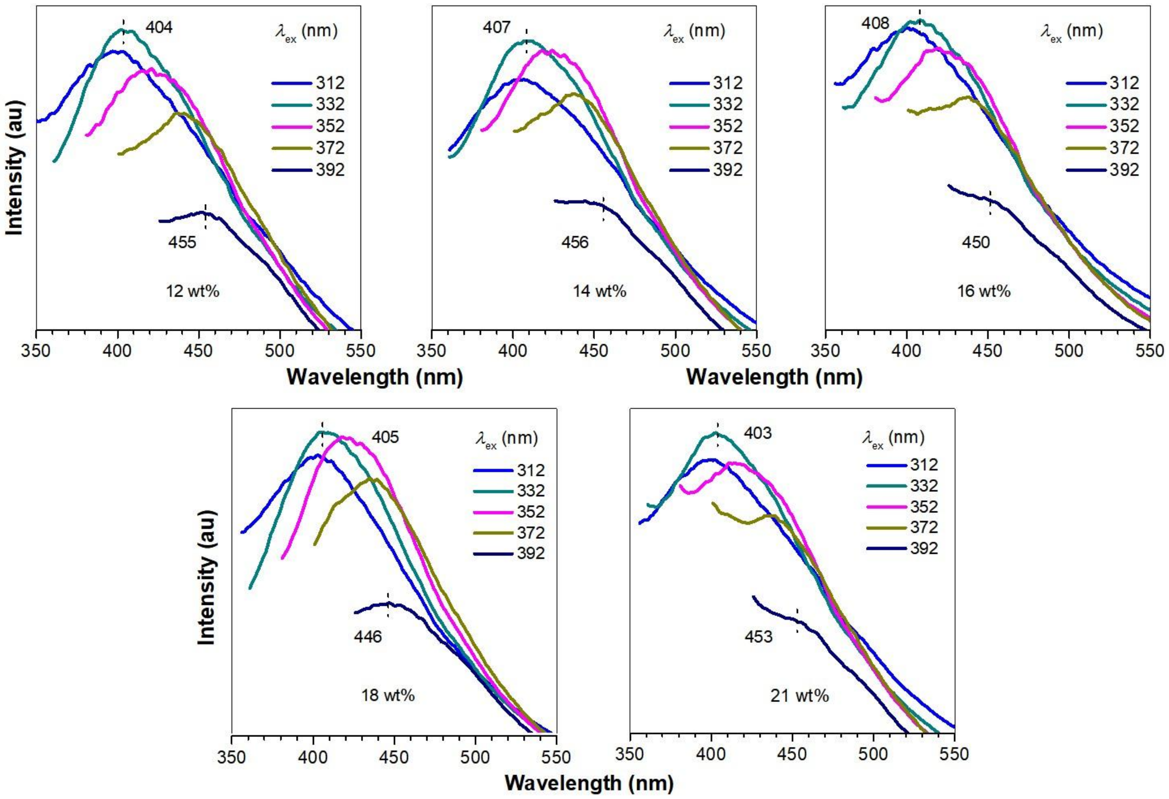

Figure S4. The emission spectra of samples (12, 14, 16, 18 and 21 wt%) at different excitation.

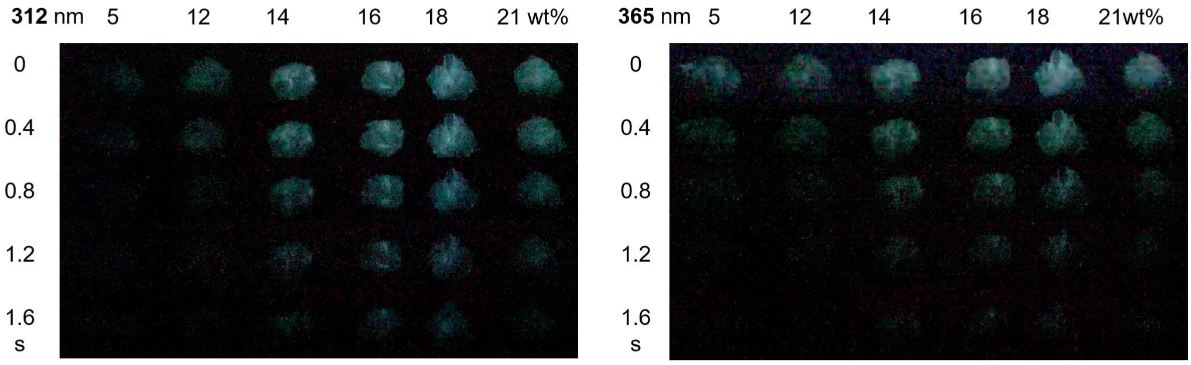

Figure S5. Photographs of samples after ceasing the 312 and 365 nm irradiation at ambient conditions.

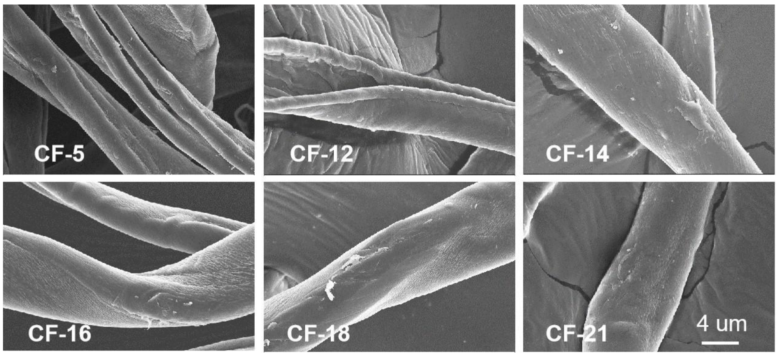

Figure S6. SEM images of CF surface with different crystallinity (scale bar=4 um).

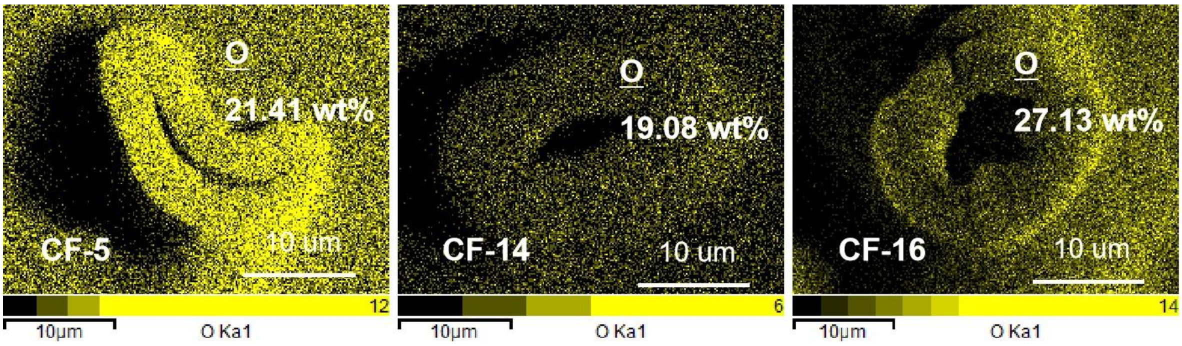

Figure S7. SEM mapping images of oxygen of CF5, CF-14 and CF-16.

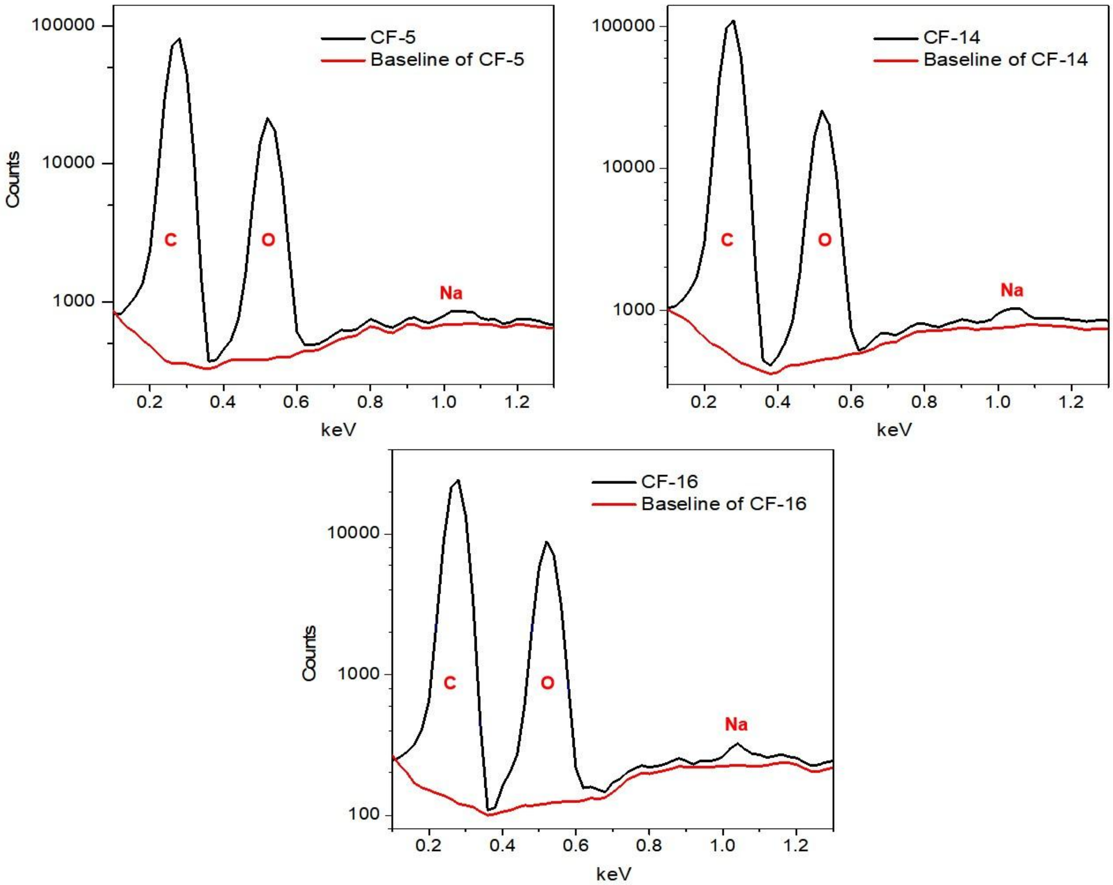

Figure S8. EDX mapping spectra and background curves of three CF samples.

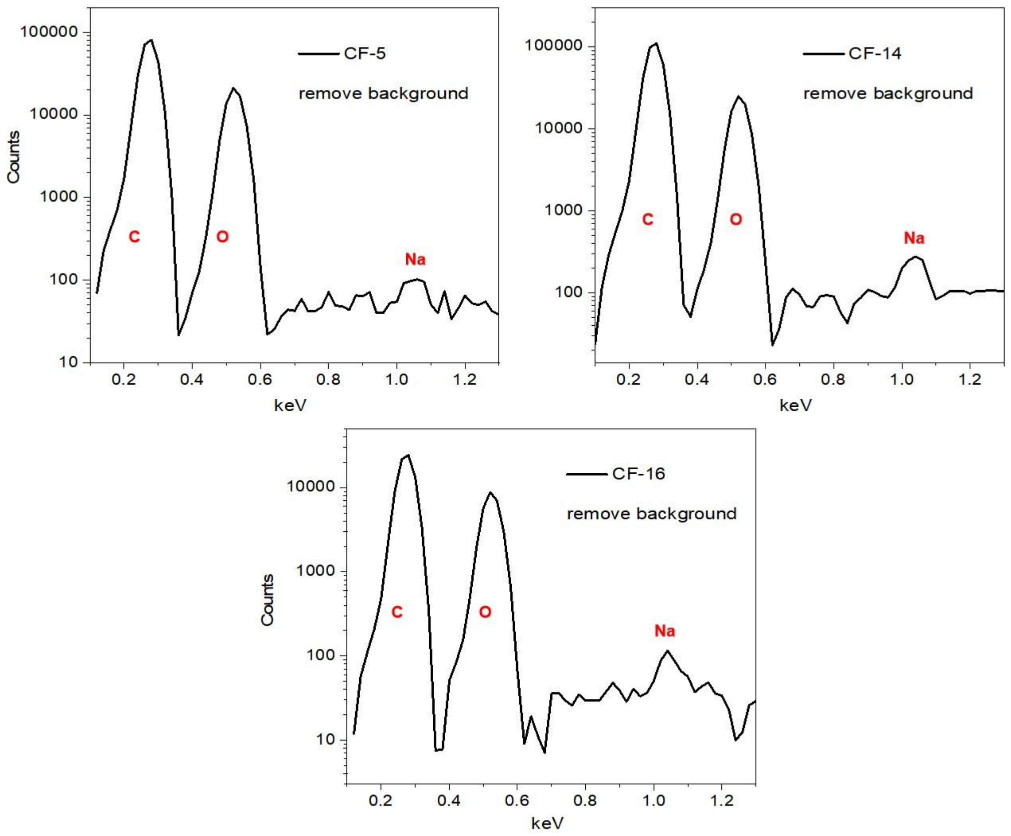

Figure S9. EDX mapping spectra of three CF samples after background subtraction.

Table S2. Summary of the *p*-RTP lifetimes of samples.[a]

wt%	τ_1 [ms]	A_1 [%]	τ_2 [ms]	A_2 [%]	τ_3 [ms]	A_3 [%]	τ [ms]	$\lambda_{ex,RTP}$ [nm]	$\lambda_{em,RTP}$ [nm]
5	13.56	51.24	13.57	47.39	92.93	1.37	14.65	280	490
12	13.06	52.16	13.07	46.83	152.77	1.01	15.40	280	490
14	14.76	48.63	14.80	48.42	110.10	2.96	17.60	280	490
16	3.08	66.56	17.85	14.90	81.92	18.54	19.90	280	490
18	16.61	48.41	16.61	48.07	124.41	3.52	20.40	280	490
21	14.75	49.27	14.75	48.61	123.35	2.12	17.05	280	490

[a] All measurements were conducted at ambient conditions. $\lambda_{ex,RTP}$ = excitation wavelength used for the lifetime measurement; $\lambda_{em,RTP}$ = monitored emission wavelength. $\tau = (A_1\tau_1^2 + A_2\tau_2^2 + A_3\tau_2^3)/(A_1\tau_1 + A_2\tau_2 + A_3\tau_3)$.

Table S3. Integration range, integration area and content of carbon, oxygen and Na elements in EDX mapping energy spectra

Samples	CF-5	CF-14	CF-16	Integral range
Integral area (C)	4945.69	6739.41	1492.56	0.1~0.36 keV
C[%]	78.41	80.60	72.39	
Integral area (O)	1350.15	1595.65	559.31	0.36~0.62 keV
O[%]	21.41	19.08	27.13	
Integral area (Na)	11.73	26.41	10.02	0.96~1.1 keV
Na[%]	0.19	0.32	0.49	
